# Whole Grain Qingke Attenuates High-Fat Diet-Induced Obesity in Mice With Alterations in Gut Microbiota and Metabolite Profile

**DOI:** 10.3389/fnut.2021.761727

**Published:** 2021-12-07

**Authors:** Xipu Li, Jingqi Suo, Xinguo Huang, Huifen Dai, Hongwu Bian, Muyuan Zhu, Weiqiang Lin, Ning Han

**Affiliations:** ^1^Institute of Genetic and Regenerative Biology, Key Laboratory for Cell and Gene Engineering of Zhejiang, College of Life Sciences, Zhejiang University, Hangzhou, China; ^2^The Fourth Affiliated Hospital of Zhejiang University School of Medicine, Yiwu, China; ^3^Institute of Translational Medicine, The Fourth Affiliated Hospital, Zhejiang University School of Medicine, Hangzhou, China

**Keywords:** whole grain, obesity, gut microbiota, fecal metabolites, *CYP7A1*, FXR, tryptophan metabolism, unsaturated fatty acids

## Abstract

Whole grain Qingke (WGQK) displays anti-obesity and lipid-lowering properties; however, the underlying mechanism remains elusive. This study investigated the alteration of gut microbiota composition and metabolite profile induced by WGQK intervention in mice through the integration of 16S ribosomal RNA (rRNA) sequencing and an untargeted metabolomics study. C57BL/6J male mice were fed a normal control diet (NC), high-fat diet (HFD), and HFD plus 30% WGQK (HFD+QK) for 16 weeks. The WGQK intervention decreased body weight gain, glucose tolerance, and serum lipid levels, and alleviated liver function damage induced by HFD. Moreover, WGQK changed gut microbiota composition and enriched specific genera such as *Akkermansia, Bifidobacterium*, and *Lactobacillus*. Fecal metabolomics analysis indicated that WGQK enhanced the abundance of tryptophan metabolism-related metabolites (indole, 3-indoleacetic acid, indole acetic acid (IAA), 5-hydroxyindole-3-acetic acid), histidine metabolism-related metabolites (histamine), and some unsaturated fatty acids (oleic acid, 9,10-dihydroxy-12Z-octadecenoic acid, and alpha-linolenic acid). Spearman correlation analysis revealed that these metabolites were negatively correlated with obesity-related parameters and positively correlated with the gut genera enriched by WGQK. Moreover, WGQK promoted the expression of Cholesterol 7α-hydroxylase *(CYP7A1)* responsible for primary bile acids production, accompanied by a decline in intestinal *FXR*-*FGF15* expression levels. The transcript levels of two genes associated with lipogenesis, such as lipid fatty acid synthase (*FAS)* and acetyl-CoA carboxylase (*ACC)* were also decreased in the HFD+QK group. Overall, our results suggest interactions between gut microbial shifts and host amino acid/lipid metabolism, and shed light on the mechanisms underlying the anti-obesity effect of WGQK.

## Introduction

Obesity has become an important global health issue, defined by the WHO as excessive fat accumulation that might damage health; it is also related to a reduction in life expectancy. According to statistics, nearly 39% of the world population is overweight, and 13% of adults are obese (1). Obesity is a chronic progressive disease that can greatly increase the risk of type 2 diabetes, fatty liver, hypertension, myocardial infarction, stroke, and certain types of cancers (1, 2). Exercise and limiting calorie intake have been implicated as effective ways to lose weight. However, for most people, it is difficult to persevere; only a few obese patients successfully persist in required lifelong diet and exercise plans (3). Therefore, dietary interventions may be a potential way to control obesity and maintain health.

Whole grains are mainly defined as whole ground split or flaky caryopsis. Its main anatomical components are the starchy endosperm, bran, and germ, and they exist in the same relative proportions as in the intact caryopsis (4). Whole grains contain many bioactive compounds that are beneficial to human health. Barley (*Hordeum vulgare L*.) is the fourth most produced cereal globally and has the highest dietary fiber content (5). In addition, barley grains contain a large number of phytochemicals such as β-glucan, phenolic acids, tocols, phytosterols, and folate, which can decrease the risk of chronic diseases (6, 7). Qingke is a hull-less barley cultivar that grows in the highland area and has been used as a primary staple food in the Qinghai Tibetan Plateau, China. Compared with other cultivated barley, hull-less barley has more advantages in processing and food applications (8). Furthermore, some studies have reported that Qingke and barley have hypolipidemic effects and can decrease serum glucose levels and improve oxidation resistance and obesity in animal models (9, 10). However, the mechanisms underlying these effects remain unclear.

The effects of diet on host metabolism, energy homeostasis, and host immune response are thought to be partly achieved by regulating the gut microbiota (11). Accumulating data have demonstrated that the gut microbiota is altered in individuals with obesity and type 2 diabetes, suggesting a direct link between gut microbiota and metabolic diseases (12, 13). A previous study showed that conventionally raised animals had 42% more total body fat than germ-free (GF) mice, even though the GF mice consumed 29% more chow (14). Another study revealed that transplantation of cecal contents from genetically obese (ob/ob) mice resulted in an even greater increase in body fat (15). The increase of the relative abundance of Firmicutes, Archaea, and decrease the Bacteroidetes have been reported to associate with obesity (12, 16). Moreover, the increase of *Akkermansia, Bifidobacterium*, and *Lactobacillus* have been implicated in alleviating obesity and hyperlipidemia (17–19).

The physiological effect of gut microbes on host metabolism has been thought to associate with various metabolites produced by gut microbiota, including short-chain fatty acids (SCFAs) and secondary bile acids (20). The microbiota in the lower intestinal ferment polysaccharides into SCFAs. The SCFAs can provide energy to the cell of colon and attenuate inflammation (21). A randomized clinical study, together with fecal shotgun metagenomics, showed that a select group of SCFA-producing strains was promoted by dietary fibers to alleviate type 2 diabetes, suggesting a novel ecological approach for managing T2DM (22). Another important function of gut microbes is the modification of primary bile acids into secondary bile acids, which is important for regulating glucose homeostasis *via* farnesoid X receptor (FXR) (23). On the other hand, BAs can modulate the gut microbiota composition through the bile-sensitive and bile-metabolizing bacteria and through FXR-mediated transcription of antimicrobial agents (24, 25). Overall, the cross-talk among host metabolism, gut microbiota and metabolites plays a key role in regulating energy harvest, lipid metabolism as well as cholesterol and BA homeostasis. Previous studies revealed that intake of whole grain barley (or Qingke), barley-containing food, or barley-derived β-Glucan modified gut microbiota structure, predominantly induced a group of short-chain fatty acid producers, which might be beneficial for host health (9, 26, 27). Nonetheless, the link between changes in gut microbiota structure and host glucose and lipid metabolism is still not fully understood.

In this study, we investigated the effects of the intake of whole grain Qingke on glucose and lipid metabolism in a mouse model of high-fat diet (HFD)-induced obesity. We revealed the relationship between bacterial composition and the complex metabolic network in the gut by integrated analysis of 16S ribosomal RNA (rRNA) gene sequencing and untargeted metabolomics study. We characterized some WGQK-dependent specific gut bacteria and metabolites, which might be helpful for our understanding of the mechanisms underlying the anti-obesity effect of WGQK.

## Materials and Methods

### Animals and Diet Designs

The animal study was reviewed and approved by the Institutional Animal Care and Use Committee of the Laboratory Animals Center at Zhejiang University, Hangzhou, China (No. ZJU20210160). A total of 15 male C57BL/6 mice (weighting 21–22 g, 6-week-old) were purchased from Shanghai Silaikang Experimental Animal Co., Ltd. (Shanghai, China). The mice were maintained in a constant temperature (22–24°C) environment with a 12 h light/dark cycle. After 1 week of acclimation, they were randomly allocated into three groups (*n* = 5/group): the NC group fed with low-fat D12450J (10% fat) diet, HFD group fed with high-fat D12492 (60% fat) diet, the HFD+QK group fed with high-fat D12492 plus 30% whole grain Qinke. The compositions of the experimental diets are shown in Table 1. The whole grain Qingke diet was formulated by replacing the components of the HFD diet with whole grain Qingke as the source of carbohydrates, protein, and fat, to maintain an approximately equivalent calorific value of the two diets. This study used the Qingke variety “Kunlun 14” (Xinjiang NiuWangGong Grain Cooperatives, Xinjiang, China). The seeds contain the following ingredients: carbohydrates 74.4%, protein 10.7%, and fat 2.3%. The seeds were ground to powder; no mesh was used in order to keep all the nutrients. Mice were fed the above-mentioned experimental diets for 16 weeks and received food and water *ad libitum*.

**Table 1:** Composition of experimental diets.

	**NC**		**HFD**		**HFD + WGQK**	
**Ingredient**	**g/kg**	**Kcal**	**g/kg**	**Kcal**	**g/kg**	**Kcal**
Casein,80 mesh	200	800	258.46	1,033.84	222.75	891.00
L-Cystine	3	12	3.8769	15.5076	3.88	15.52
Corn starch	506.2	2,024.8	__	__	__	__
Maltodextrin 10	125	500	161.54	646.15	__	__
Sucrose	77.8	311.2	88.91	355.64	__	__
Cellulose, BW200	50	__	64.62	__	59.31	__
Soybean oil	25	225	32.31	290.77	24.21	217.89
Lard	20	180	316.61	2,849.52	316.6	2,849.4
Mineral mix, S10026	10	__	12.92	__	12.92	__
DiCalcium phosphate	13	__	16.80	__	16.80	__
Calcium carbonate	5.5	__	7.11	__	7.11	__
Potassium citrate,1**·**H_2_O	16.5	__	21.32	__	21.32	__
Vitamin mix, V10001	1	4	12.92	51.69	12.92	51.69
Choline bitartrate	2	__	2.58	__	2.58	__
FD C blue dye	0.01	__	0.06	__	__	__
WGQK	__	__	0.00	__	300	1,097.13
Total	1,055.1	4,057	1,000.05	5,243.12	1,000.40	5,122.62

*The standards of energy determination were 9 kcal/g of fat, and 4 kcal/g of protein and carbohydrate. The nutrients content of WGQK: carbohydrates 74.4%, protein 10.7%, fat 2.3%*.

Bodyweight and food intake per cage were measured weekly. Glucose and insulin tolerance were analyzed at the 14th and 15th weeks, respectively. The feces were collected at the 16th week. Serum was collected from the eyes just before the mice were executed, liver and colon samples were harvested after the mice died. All samples were stored at −80°C until further analysis.

### Glucose and Insulin Tolerance Test

For GTT, mice were fasted for 16 h and intraperitoneally injected with D-glucose in sterile water (2 g/kg), then, blood samples were collected from the tip of the tail vein at 0, 30, 60, 90, 120 min after injection for measurement of blood glucose using Yuwell glucometer and glucose strips (YuYue Medical equipment and supply Co., Ltd., JiangSu, China). For ITT, mice were fasted for 6 h and injected with human insulin in saline (1 U/kg BW), then, blood glucose was determined at the time points of 0, 30, 60, and 90 min after injection (28).

### Serum Biochemical and Liver Histological Analysis

The levels of glutamic-pyruvic transaminase (ALT), glutamic oxalacetic transaminase (AST), low-density lipoprotein cholesterol (LDL-C), high-density lipoprotein cholesterol (HDL-C), total cholesterol (TC), and total triglyceride (TG) in serum samples were determined using an LW C400 Mindray automatic biochemical analyzer (Shenzhen Lanyun Medical Equipment Co., Ltd., Shenzhen, China).

For the liver histological study, fresh liver tissue was fixed in 4% (v/v) formaldehyde solution for 48 h, embedded in paraffin wax, sectioned, and stained with hematoxylin and eosin (H&E) and Oil Red O (ORO). Histological images were obtained using an optical microscope (Nikon, Japan). For ORO stain, five different visual fields were selected and the relative Oil Red O-stained area of the cells was quantified using Image J.

### Gut Microbiota Analysis

#### DNA Extraction

At the 16th week of the animal trial, fresh feces were collected from each mouse and stored at −80°C until analysis. DNA from feces was extracted using the E.Z.N.A. ®Stool DNA Kit (D4015, Omega, Inc., USA) according to the manufacturer's instructions. The reagent, which was designed to uncover DNA from trace amounts of sample, has been shown to be effective for the preparation of DNA from most bacteria. Nuclear-free water was used as the blank. The total DNA was eluted in 50 μL of elution buffer and stored at −80°C until PCR was performed by LC-Bio Technology Co., Ltd., Hang Zhou, Zhejiang Province, China.

#### PCR Amplification and 16S rDNA Sequencing

The 5' ends of the primers were tagged with specific barcodes per sample and sequenced using universal primers. PCR amplification was performed in a total volume of 25 μL reaction mixture containing 25 ng of template DNA, 12.5 μL PCR Premix, 2.5 μL of each primer, and PCR-grade water to adjust the volume. The PCR conditions used to amplify the prokaryotic 16S fragments consisted of: initial denaturation at 98°C for 30 s; 32 cycles of denaturation at 98°C for 10 s, annealing at 54°C for 30 s, and extension at 72°C for 45 s; and a final extension at 72°C for 10 min. PCR products were confirmed by 2% agarose gel electrophoresis. Throughout the DNA extraction process, ultrapure water, instead of a sample solution, was used as a negative control, to exclude the possibility of false-positive PCR results. The PCR products were purified using AMPure XT beads (Beckman Coulter Genomics, Danvers, MA, USA) and quantified using Qubit (Invitrogen, USA). The amplicon pools were prepared for sequencing, and the size and quantity of the amplicon library were assessed on an Agilent 2100 Bioanalyzer (Agilent, USA) and with the Library Quantification Kit for Illumina (Kapa Biosciences, Woburn, MA, USA), respectively. The libraries were sequenced using the NovaSeq PE250 platform.

#### Data Analysis

All samples from each group (*n* = 5) were sequenced on an Illumina NovaSeq platform according to the manufacturer's recommendations, provided by LC-Bio. Paired-end reads were assigned to samples based on their unique barcodes and truncated by cutting off the barcode and primer sequences. Paired-end reads were merged using FLASH. Quality filtering of the raw reads was performed under specific filtering conditions to obtain high-quality clean tags according to fqtrim (v0.94). Chimeric sequences were filtered using the Vsearch software (v2.3.4). After dereplication using DADA2, we obtained a feature table and feature sequence. Alpha diversity and beta diversity were calculated by normalizing to the same sequences randomly. Then, according to the SILVA (release 132) classifier, the feature abundance was normalized using the relative abundance of each sample. Alpha diversity and beta diversity were calculated using QIIME2, and graphs were drawn using the R package. BLAST was used for sequence alignment, and the feature sequences were annotated with the SILVA database for each representative sequence.

### Untargeted Metabolomics Study

#### Metabolite Extraction

The collected samples (5 mice per group) were thawed on ice, and metabolites were extracted from 20 μL of each sample by using 120 μL of pre-cooled 50% methanol buffer. The mixture of metabolites was vortexed for 1 min, incubated for 10 min at room temperature (18–25°C), and stored at −20°C overnight. The mixture was centrifuged at 4,000 × g for 20 min, and the supernatant was transferred to 96-well plates. The samples were stored at – 80°C before LC-MS analysis. Pooled quality control (QC) samples were prepared by combining 10 μL of each extraction mixture.

#### LC-MS Analysis

All samples from each group (*n* = 5) were analyzed using a TripleTOF 5600 Plus high-resolution tandem mass spectrometer (SCIEX, Warrington, UK) with both positive and negative ion modes. Chromatography was performed for the separation of the metabolites by using an ultra-performance liquid chromatography (UPLC) system (SCIEX, UK). An ACQUITY UPLC T3 column (100 ^*^ 2.1 mm, 1.8 μm, Waters, UK) was used for reversed-phase separation. The mobile phase consisted of solvent A (water, 0.1% formic acid) and solvent B (Acetonitrile, 0.1% formic acid). The gradient elution conditions were as follows with a flow rate of 0.4 ml/min: 5% solvent B for 0–0.5 min; 5–100% solvent B for 0.5–7 min; 100% solvent B for 7–8 min; 100–5% solvent B for 8–8.1 min; and 5% solvent B for 8.1–10 min. Column temperature was maintained at 35°C.

The Triple TOF 5600 Plus system was used to detect metabolites eluted from the column. The curtain gas pressure was set at 30 psi and the ion source gas 1 and gas 2 pressures were set at 60 psi. For the positive-ion mode, the ion spray floating voltage was set at 5 kV. For the negative-ion mode, it was set at −4.5 kV. MS data were acquired in IDA mode. The TOF mass range was 60–1,200 Da. Survey scans were acquired every 150 ms, and as many as 12 product ion scans were collected if the threshold of 100 counts/s was exceeded with a 1 + charge state. The total cycle time was fixed at 0.56 s. Four-time bins were summed for each scan at a pulse frequency of 11 kHz by monitoring the 40 GHz multichannel TDC detector with four-anode/channel detection. Dynamic exclusion was set for 4 s.

#### Metabolomics Data Processing

The acquired LC-MS data were pre-treated using the XCMS software. Raw data files were converted into mzXML format and then processed using the XCMS, CAMERA, and metaX toolbox included in the R software. Each ion was identified using comprehensive information on the retention time and m/z. The intensity of each peak was recorded, and a three-dimensional matrix containing arbitrarily assigned peak indices (retention time –m/z pairs), sample names (observations), and ion intensity information (variables) was generated. Then, the information was matched to in-house and public databases. The open access databases, KEGG and HMDB, were used to annotate the metabolites by matching the exact molecular mass data (m/z) to those from the database within a threshold of 10 ppm. The peak intensity data were further pre-processed using metaX software. Features that were detected in <50% of QC samples or 80% of test samples were removed, and values for missing peaks were extrapolated with the k-nearest neighbor algorithm to further improve the data quality. PCA was performed to detect the outliers and batch effects by using the pre-processed dataset. The QC-based robust LOESS signal correction was fitted to the QC data with respect to the order of injection to minimize signal intensity drift over time. In addition, the relative standard deviations of the metabolic features were calculated for all QC samples, and those with standard deviations >30% were removed. The group datasets were normalized before analysis was performed. Data normalization was performed on all samples by using a probabilistic quotient normalization algorithm.

#### RNA Extraction, Reverse Transcription, and RT-PCR Analysis

Total RNA from liver and colon was extracted using RNAiso Plus (TaKaRa, Dalian, China), and 2 μg was used for first-strand cDNA synthesis using oligo (dT) primers and a Super-Script III RT kit (TaKaRa, Dalian, China). Quantitative real-time PCR (qRT-PCR) was performed on a Mastercycler Ep Realplex2 system (Eppendorf, Hamburg, Germany) using the SYBR PrimeScript RT Reagent Kit (Perfect Real Time, TaKaRa, China). The sequences of primer pairs are listed in Supplementary Table S1. Relative transcript levels were calculated using the ΔΔCt method, and the 18S rRNA gene was used as a reference.

### Statistics

All data are presented as the mean ± SD, and comparisons between two groups or time points were performed using an unpaired two-tailed *t*-test. Different letters and asterisks indicate statistical significance, letters: different superscript letters of any two groups indicate significant differences (*p* < 0.05); asterisks: ^*^p < 0.05, ^**^p < 0.01. The correlation between host parameters and gut microbiota or metabolites was determined using Spearman correlation analysis.

## Results

### WGQK Alleviated HFD-Induced Body Weight Gain and Type 2 Diabetes Associated Symptom

**Figure 1:**
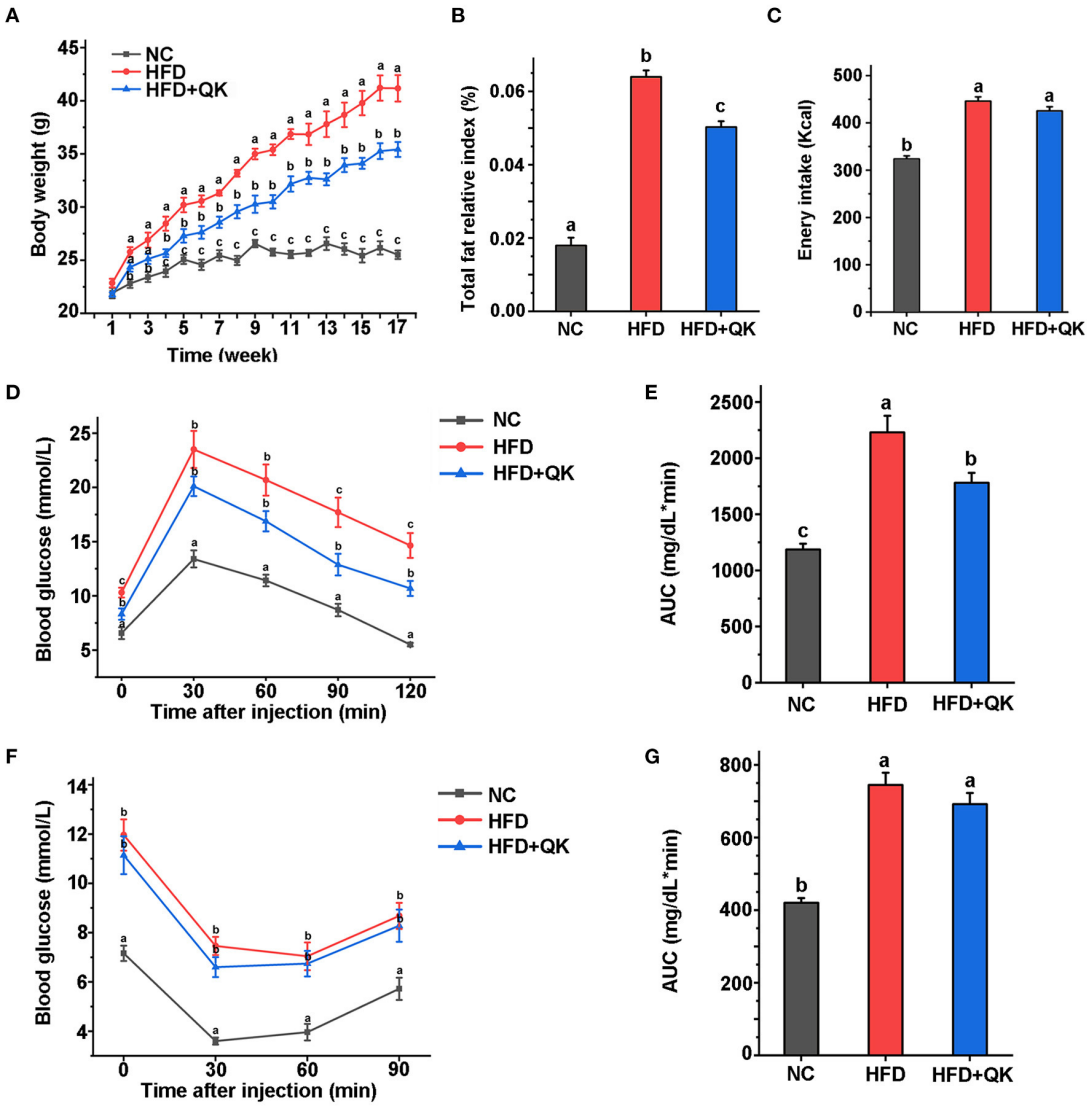
Effects of the intake of WGQK on obesity and diabetes-related parameters. **(A)** Mean weekly body weight of the NC, HFD, and HFD+QK groups (*n* = 5); **(B)** Mean total fat relative index (total fat weight/body weight) of each group after 16 weeks of the diet intervention; **(C)** Mean weekly energy intake of each group; **(D)** Blood glucose levels at different time points during glucose tolerance test (GTT); **(E)** Mean areas under the curve (AUC) of GTT; **(F)** Mean blood glucose levels during insulin tolerance test (ITT); **(G)** Mean area under the curve (AUC) of ITT. Data are presented as mean ± SD (*n* = 5). Significant difference was determined using an unpaired two-tailed *t*-test, different superscript letters of any two means represent significant differences (*p* < 0.05).

Male C57BL/6 mice were fed either a normal diet (NC group) or a high-fat diet (HFD group) or high-fat plus whole grain Qingke (HFD+QK group) for 16 weeks from 7 weeks of age. After high-fat diet treatment, both HFD and HFD+QK mice exhibited significantly increased body weight compared with the NC group, and WGQK intervention inhibited high-fat diet-induced weight gain (Figure 1A). Furthermore, HFD increased the total fat mass, while WGQK reduced the total fat weight compared to the HFD group (Figure 1B). During the 16-week trial period, the total energy intake was monitored, showing that the energy consumed by the NC group was significantly lower than the other two groups. No significant difference in energy consumed was observed between the HFD and HFD+QK groups (Figure 1C). This indicates that the decreased body weight and total fat relative index gain in the HFD+QK group were not due to decreased food intake.

Diabetes is the most common complication of obesity (1). To measure the body's ability to regulate blood glucose, we conducted a glucose tolerance test (GTT)- and insulin tolerance test (ITT). The serum GTT assay showed that the blood glucose of HFD mice was higher than that of NC mice at every time point tested (*p* < 0.05), and the HFD+KQ mice had significantly lower blood glucose at two- time points (90 and 120 min) and the area under the curve (AUC), compared with the HFD mice (*p* < 0.05; Figures 1D,E). The ITT assay showed a similar trend, but did not reach statistical significance (Figures 1F,G). These results indicate that WGQK feeding alleviated the HFD-induced decline in glucose utilization ability.

### WGQK Affected Blood Lipid Profiles and Liver Function

**Figure 2:**
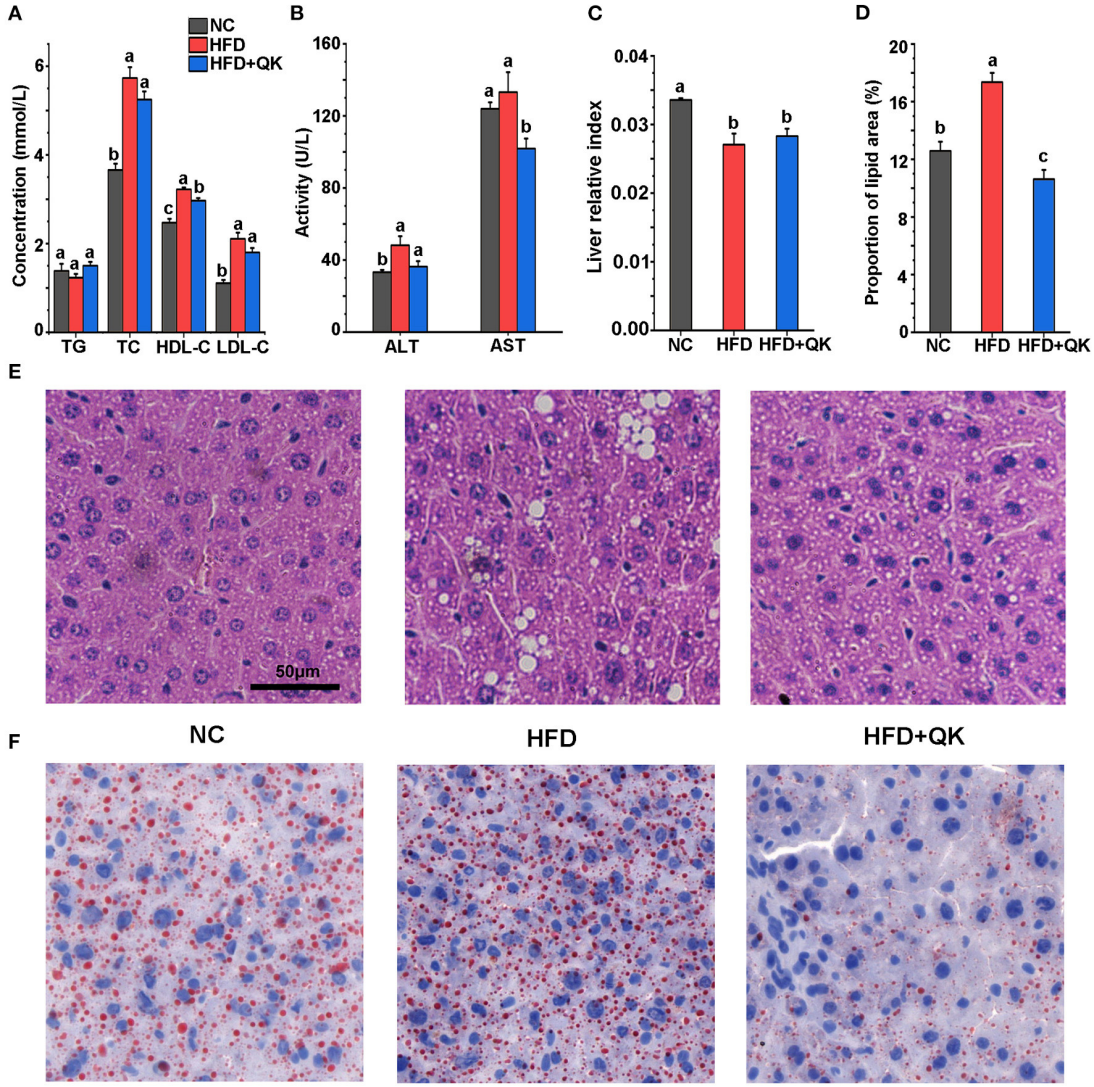
Effects of WGQK on serum lipid parameters, liver function indicators, and histology. **(A)** Mean concentration of total triglyceride (TG), total cholesterol (TC), high-density lipoprotein cholesterol (HDL-C), and low-density lipoprotein cholesterol (LDL-C) in the serum mice of each group; **(B)** Mean serum glutamic-pyruvic transaminase (ALT) and glutamic oxalacetic transaminase (AST) levels; **(C)** Liver relative index of each group; **(D)** Mean proportion of lipid area of five different visual fields of each group (calculated by Image J); **(E)** Representative photomicrographs of H&E-stained sections of the liver of each group mice showing white lipid droplets (400× magnification). **(F)** Representative photomicrographs of oil-red stained sections of the liver (400× magnification), red represent lipid droplets. Data are presented as mean ± SD (*n* = 5). Significant difference was determined using an unpaired two-tailed *t*-test, different superscript letters of any two means represent significant differences (*p* < 0.05).

Obesity-related biochemical indicators of mice were measured after 16 weeks. Total cholesterol (TC), total triglyceride (TG), high-density lipoprotein cholesterol (HDL-C), and low-density lipoprotein cholesterol (LDL-C) levels in the three groups were analyzed. Both the HFD and HFD+QK groups had significantly higher TC, HDL-C, and LDL-C levels than the NC mice. The HFD+QK group had lower TC and LDL-C levels than the NC group, although the difference was not significant relative to the HFD group. Notably, we found that WGQK intervention significantly reduced HDL-C in HFD+QK mice compared with HFD mice (*p* < 0.05; Figure 2A).

We then tested the glutamic-pyruvic transaminase (ALT) and glutamic oxalacetic transaminase (AST) levels in the serum, which reflect the damage to liver cell function. HFD treatment led to increased levels of ALT, but not AST. AST levels were markedly lower in the HFD+QK group than in the HFD and NC groups (Figure 2B). In addition, the relative liver index of the HFD and HFD+QK groups was lower than the NC group (Figure 2C). By observing H&E and ORO staining of liver tissue, we found that compared with the NC mice, the HFD mice had a larger number of circular lipid droplets. WGQK treatment reduced the number and size of lipid droplets (Figures 2E,F), and the proportion of lipid area (Figure 2D), suggesting that the intake of WGQK effectively inhibited lipid accumulation in the liver tissue. These results indicated that WGQK reduced liver damage induced by HFD.

### WGQK Reduced the Richness and Diversity of Gut Microbiota

**Figure 3:**
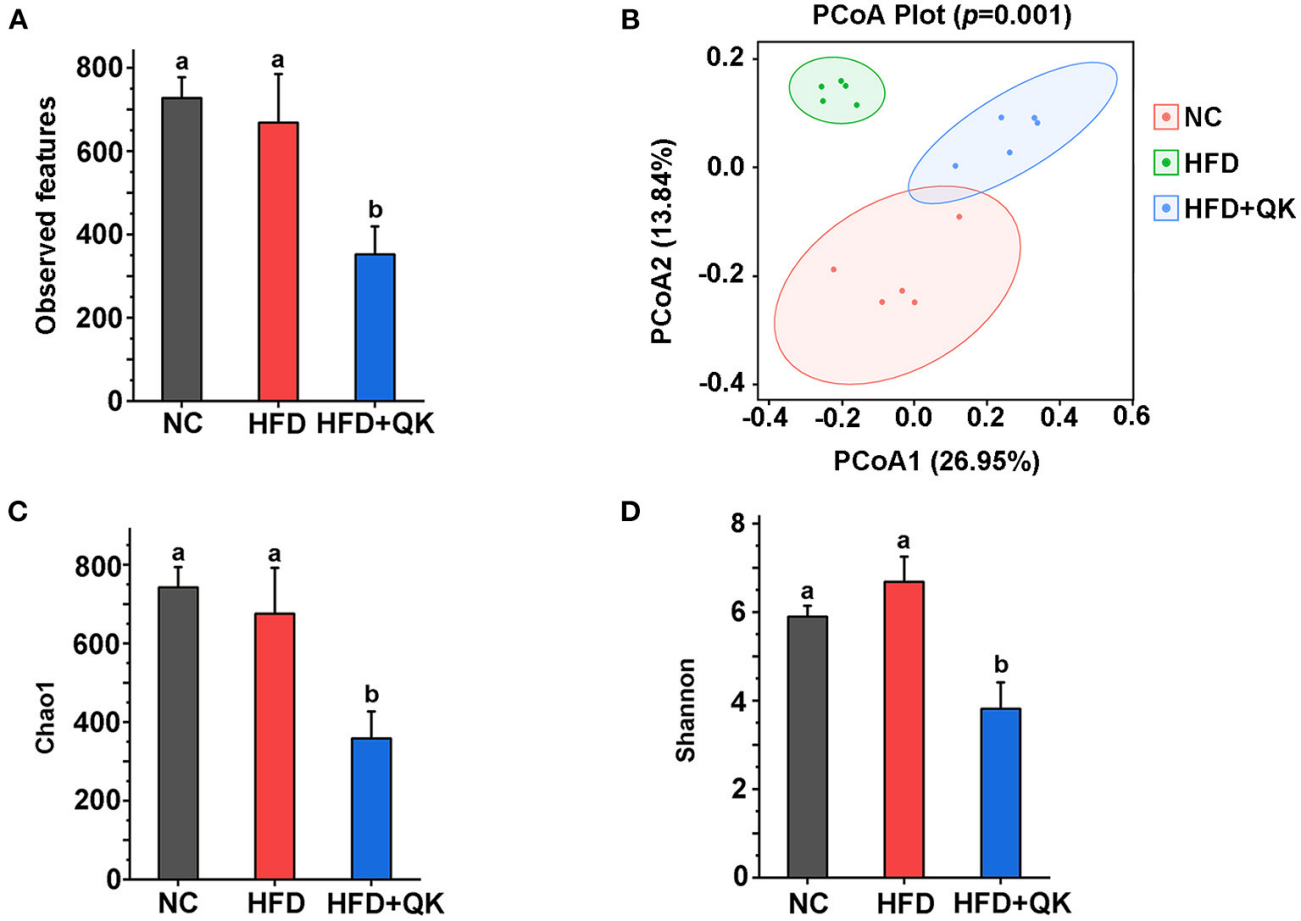
Effect of WGQK on α-diversity and β-diversity of gut microbiota in mice. **(A)** Average observed feature numbers of each group's mice; **(B)** Principal coordinates analysis score plot (PCoA) of gut microbiota among three groups; **(C,D)** Two index of α-diversity analysis, **(C)** Chao1 and **(D)** Shannon of three groups. Data are presented as mean ± SD (*n* = 5), significant difference was determined using an unpaired two-tailed *t*-test, different superscript letters of any two means represent significant differences (*p* < 0.05).

The overall changes in gut microbiota upon HFD and WGQK treatment were evaluated by sequencing the 16S rRNA genes of fecal samples isolated from the NC, HFD, and HFD+QK groups. After sequence optimization and quality filtering, a total of 3,341, 3,636, and 1,758 feature sequences were generated for the NC, HFD, and HFD + QK groups, respectively. The number of observed features in the HFD group was lower than in the NC group, and WGQK treatment further decreased the number of features (Figure 3A).

β-Diversity measures inter-community diversity. UniFrac distance-based principal coordinate analysis (PCoA) was used to reveal distinct clustering of intestinal microbial communities in each experimental group. Remarkable changes in the microbiota community structure were mediated by both HFD and WGQK (Figure 3B). Alpha diversity represents the gut microbial richness within the sample. Indices of alpha diversity, such as Shannon and Chao1, were analyzed (Figures 3C,D). The Shannon and Chao1 indices of HFD mice were not significantly different from those of NC mice (*p* > 0.05). In contrast, the WGQK mice had significantly lower Shannon and Chao1 indices compared to the HFD mice or NC mice (*p* < 0.05; Figures 3C,D). This indicates that HFD did not influence alpha diversity within the microbial community, whereas WGKQ reduced this diversity.

### WGQK Reshaped Gut Microbiota Composition in HDF Diet Mice

**Figure 4:**
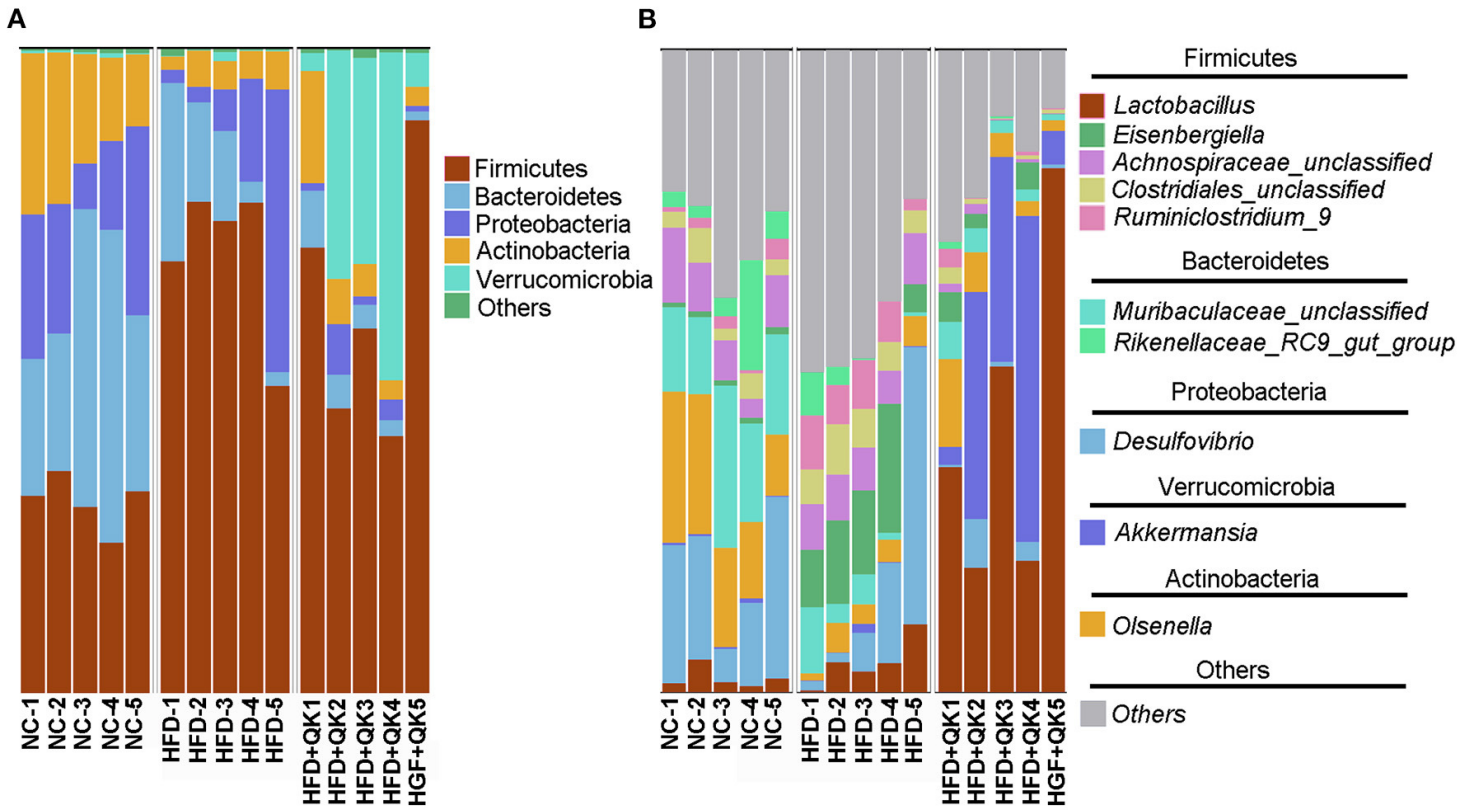
Effects of WGQK on gut microbiota composition in mice. **(A)** Phylum-level gut microbiota composition in the three mice groups. According to the relative abundance, the top 5 phyla labeled with their taxonomic names are shown. **(B)** Genus-level gut microbiota composition. Top 10 genera in the three mice groups are shown.

The composition and relative abundance of gut microbiota in the three groups were further analyzed. At the phylum level, Firmicutes was the most dominant phylum in all three mice groups. The relative abundances of the phyla Bacteroidetes, Proteobacteria, and Actinobacteria were reduced in response to HFD, both in the HFD and HFD+QK groups. In addition, the HFD group presented a lower relative abundance of Bacteroides than the other two groups. HFD feeding induced enrichment in Firmicutes, whereas WGQK decreased its relative abundance. It is worth noting that the WGQK mice presented a significantly higher abundance of Verrucomicrobia than the other two groups (Figure 4A). At the genus level, *Lactobacillus* and *Akkermansia* were more abundant in the WGQK group than in the other groups (Figure 4B).

**Figure 5:**
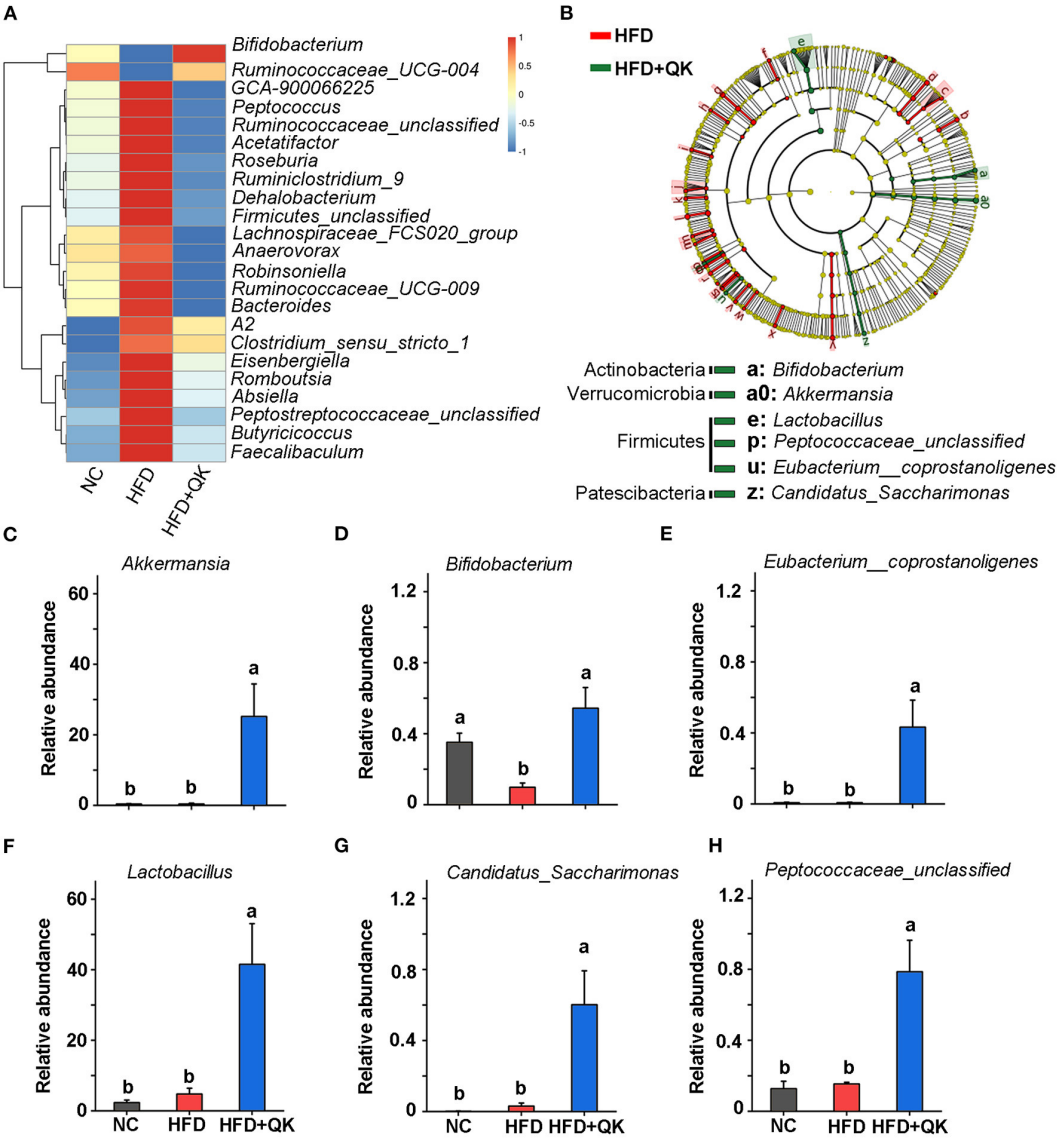
Effects of WGQK on the abundance of five key gut bacteria (genus-level). **(A)** Heat map of the significant differential genera in the HFD and WGQK mice groups; **(B)** LEfSe analysis (Kruskal-Wallis < 0.01 Wilcoxon test < 0.01, LDA score > 3) of HFD vs. HFD+QK (for a clearer display of the key bacteria, only show genus and phylum-level enrichment in the HDF+QK group); **(C–H)** The relative abundance of six key genera enriched by WGQK, **(C)** *Akkermansia*; **(D)** *Bifidobacterium*; **(E)** Eubacterium__coprostanoligenes; **(F)** *Lactobacillus Peptococcaceae_unclassified*; **(G)** *Candidatus_Saccharimonas*; **(H)** *Eubacterium__coprostanoligenes*. Data are presented as mean ± SD (*n* = 5), significant difference was determined using an unpaired two-tailed *t*-test, different superscript letters of any two means represent significant differences (*p* < 0.05).

To identify the key phylotypes of gut microbiota associated with HFD and WGQK intervention, we used the Kruskal-Wallis test with a standard of *p* < 0.05, to screen the differentially enriched genera in NC vs. HFD and HFD vs. HFD+QK. The results are shown in Supplementary Figure 1. We found that there were over 23 genera that displayed the opposite trend. Except for two of them (*Bifidobacterium* and *Ruminococcaceae_UGG-004*), which negatively responded to HFD, the remaining 21 genera were significantly promoted by the HFD, and the WGQK alleviated these trends (Figure 5A). These results indicate that WQGK intervention could restore the gut microbiota composition shifts induced by HFD.

Meanwhile, we performed LEfSe analysis of HFD+QK vs. HFD group, with a filtering threshold Kruskal-Wallis and Wilcoxon test under 0.01 and LDA score beyond 3. The results showed that WGQK specifically enriched the abundance of *Bifidobacterium, Lactobacillus, Peptococcaceae_unclassified, Eubacterium_coprostanoligenes, CandidatusSaccharimonas*, and *Akkermansia* (Figure 5B). In particular, the relative abundance of *Akkermansia* and *Lactobacillus* increased from 0. to 25.19% and from 4. to 41.49%, respectively (Figures 5C,F).

### WGQK Intervention Affects the Metabolomic Profile of the Feces

To identify how WGQK and HFD affected the metabolites of the gut microbiota in mice, the untargeted metabolome of the feces was analyzed. Two multivariate PLS-DA models with the parameters of *R*^2^ = 0.925, *Q*^2^ = −0.856 (HFD vs. NC) and *R*^2^ = 07918, *Q*^2^ = −0.9685 (HFD vs. WGQK) were constructed, indicating that the models had a good fit and could be used to evaluate the variation in metabolic profiles (Supplementary Figures 2A,B).

Significantly differential metabolite ions were identified using the standard of ratio ≥2 or ≤ 0.5, and VIP ≥ 1, as shown in the volcano plot (Supplementary Figures 2C,D). There were 200 identified metabolites among the differential ions, including 71 metabolites between the NC and HFD mice and 129 metabolites between the HFD and HFD+QK mice. Among them, 22 significantly differentially enriched metabolites existed in HFD vs. NC and WGQK vs. HFD. Interestingly, except 2-Indolecarboxylic acid, these metabolites had opposite regulatory trends in the WGQK group compared with the HFD group (Supplementary Figures 2E,F). There were 12, 9, 10, and 12 metabolites that were significantly correlated with the decrease in ALT, AST, AUC levels of GTT, and body weight gain, respectively (Supplementary Figure 3).

**Figure 6:**
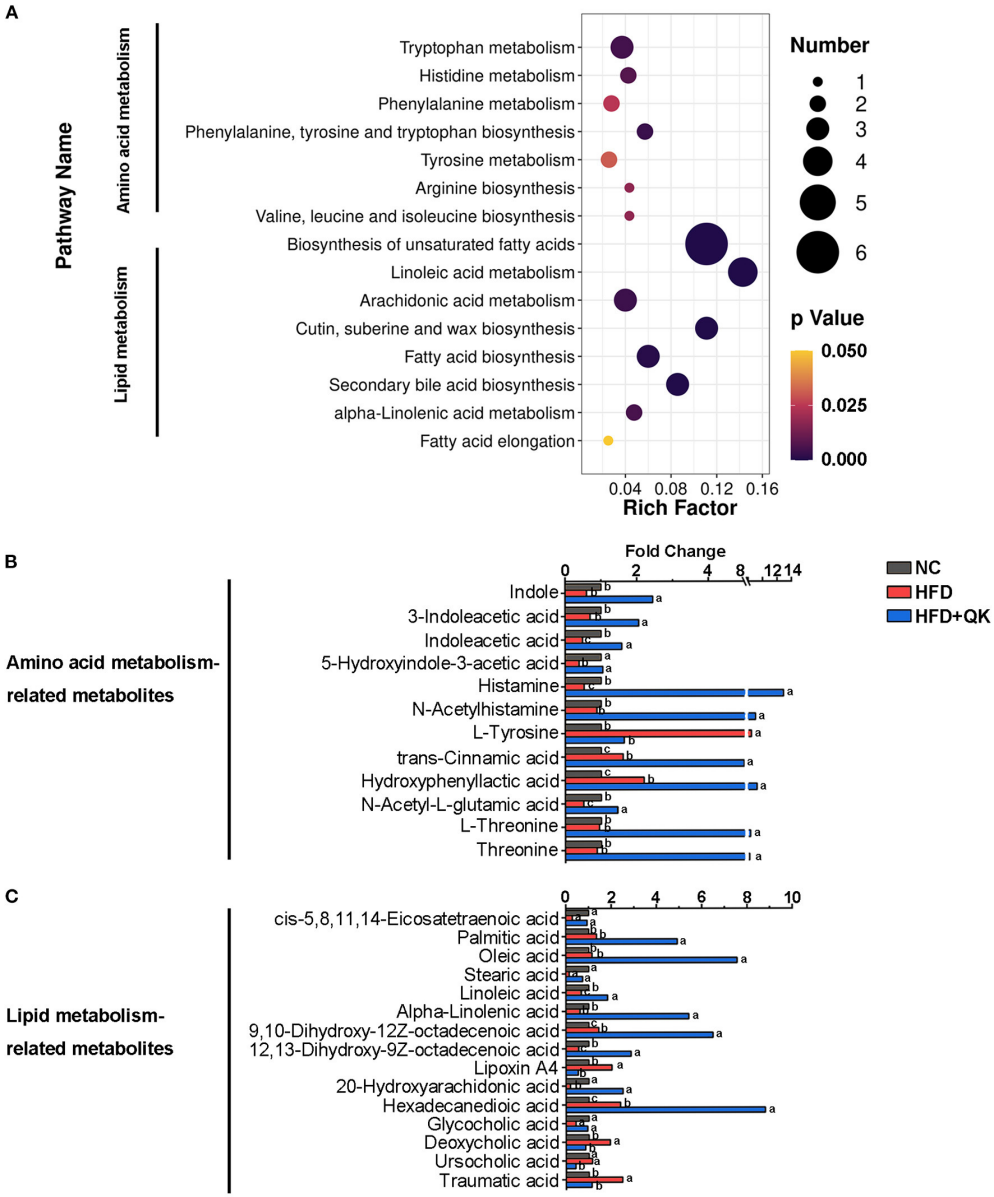
Effects of WGQK on fecal metabolites. **(A)** KEGG Enrichment Scatter Plot of Amino acid metabolism and lipid metabolism. Rich Factor= S_metabolites_Number/B_metabolites_Number, the size of the bubble indicates the amount of significantly differential metabolites which are enriched in this pathway, and the point with different gradation of color represents the scope of *p*-value. The higher value of rich factor stands for the higher degree of enrichment, and the lower *p*-value represents the more significant degree of enrichment; **(B,C)** The fold change of metabolite abundance involved in **(B)** amino acid metabolism, and **(C)** lipid metabolism.

Based on the pathway enrichment analysis, the WGQK diet mainly affected amino acid and lipid metabolism, when compared with the HFD group (Figure 6A). The fold changes in the metabolites involved in these two pathways are shown in Figures 6B,C. Among metabolites related to the amino acid pathway, nine metabolites, including histamine, L-threonine, threonine, N-acetylhistamine, N-acetyl-L-glutamic acid, and metabolites related to tryptophan metabolism [indole, 3-indoleacetic acid, indole acetic acid (IAA), and 5-hydroxyindole-3- acetic acid] were significantly enhanced in the HFD+QK group, whereas they decreased or remained unchanged in the HFD group. Two metabolites, trans-cinnamic acid and hydroxyphenyllactic acid, accumulated in both the HFD and HFD+QK mice, and the HFD+QK mice had significantly higher levels than the HFD mice. The abundance of L-tyrosine exhibited an opposite pattern, with a higher level in HFD mice, whereas it was maintained at a low level in the NC and HFD+QK mice (Figure 6B). Of the 15 metabolites associated with lipid metabolism, levels of five metabolites- palmitic acid, oleic acid, 9,10-dihydroxy-12Z-octadecenoic acid, α-linolenic acid, and hexadecanedioic acid were substantially increased by more than four times upon WGQK diet, one of which (α-linolenic acid) was decreased in HFD mice (Figure 6C). WGQK suppressed the decrease in levels of four metabolites, cis-5,8,11,14-eicosatetraenoic acid, stearic acid, 12,13–dihydroxy-9Z-octadecenoic acid, and glycocholic acid in the HFD group.

**Figure 7:**
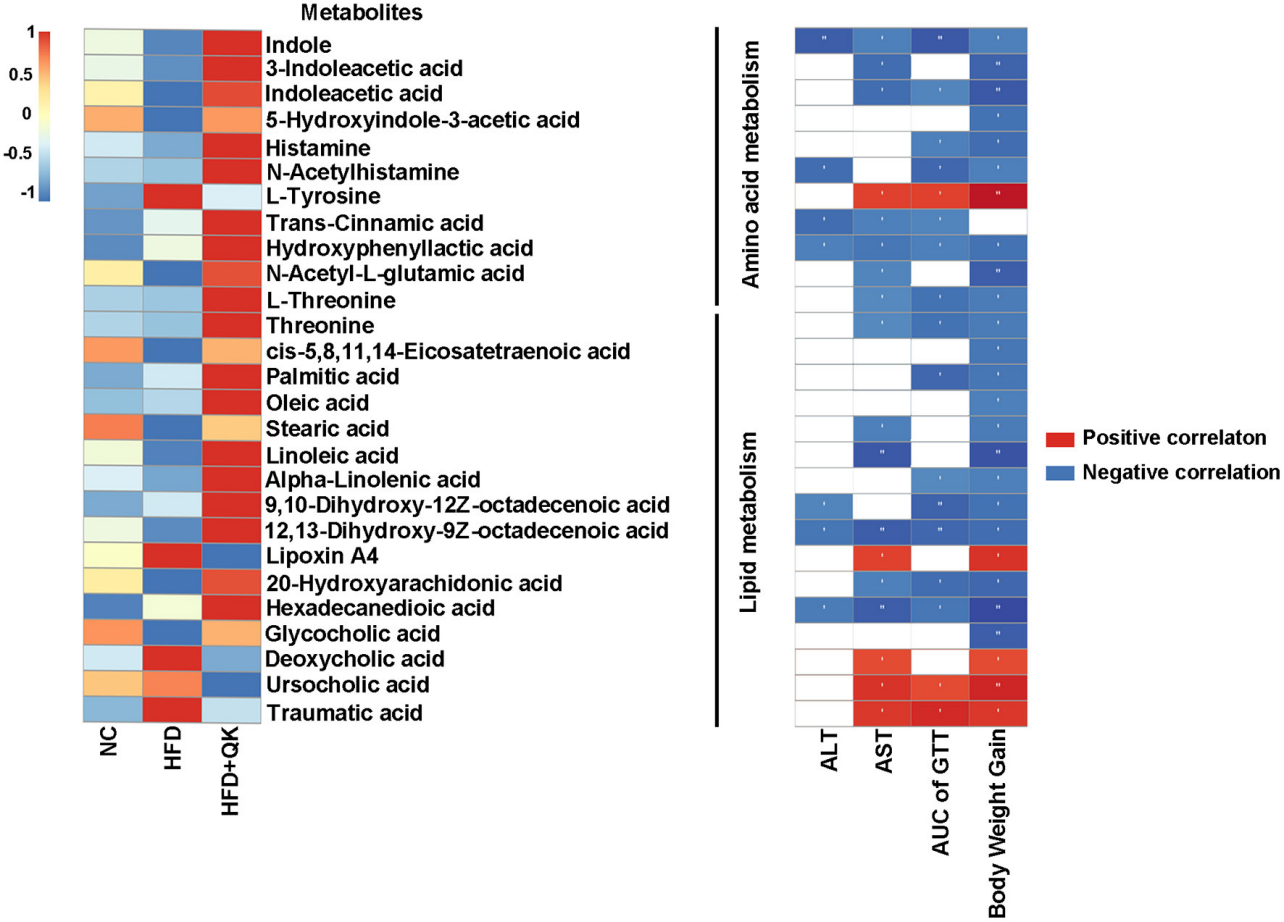
Correlation between metabolites and host parameters related to glucose and lipid metabolism. Left, heat map of metabolites involved in amino acid and lipid metabolism. Right, Spearman correlations between the abundance of these metabolites and the host parameters related to obesity. Red indicates positive correlation and black indicates negative correlation, the darker the color, the stronger the correlation, *p < 0.05, **p < 0.01.

A heat map of these metabolites and their correlations with the host parameters related to obesity are shown in Figure 7. One amino acid metabolism-related metabolite, L-tyrosine, four lipid metabolism-related metabolites (lipoxin A4, deoxycholic acid, ursocholic acid, and traumatic acid) had significant positive correlations with the host level of serum AST, glucose tolerance, and body weight gain. The metabolites of tryptophan metabolism and the unsaturated fatty acids (oleic acid, linoleic acid, alpha-linolenic acid, etc.) were negatively correlated with the host parameters (Figure 7).

### Correlation Analysis Between Gut Microbiota and Metabolites

**Figure 8:**
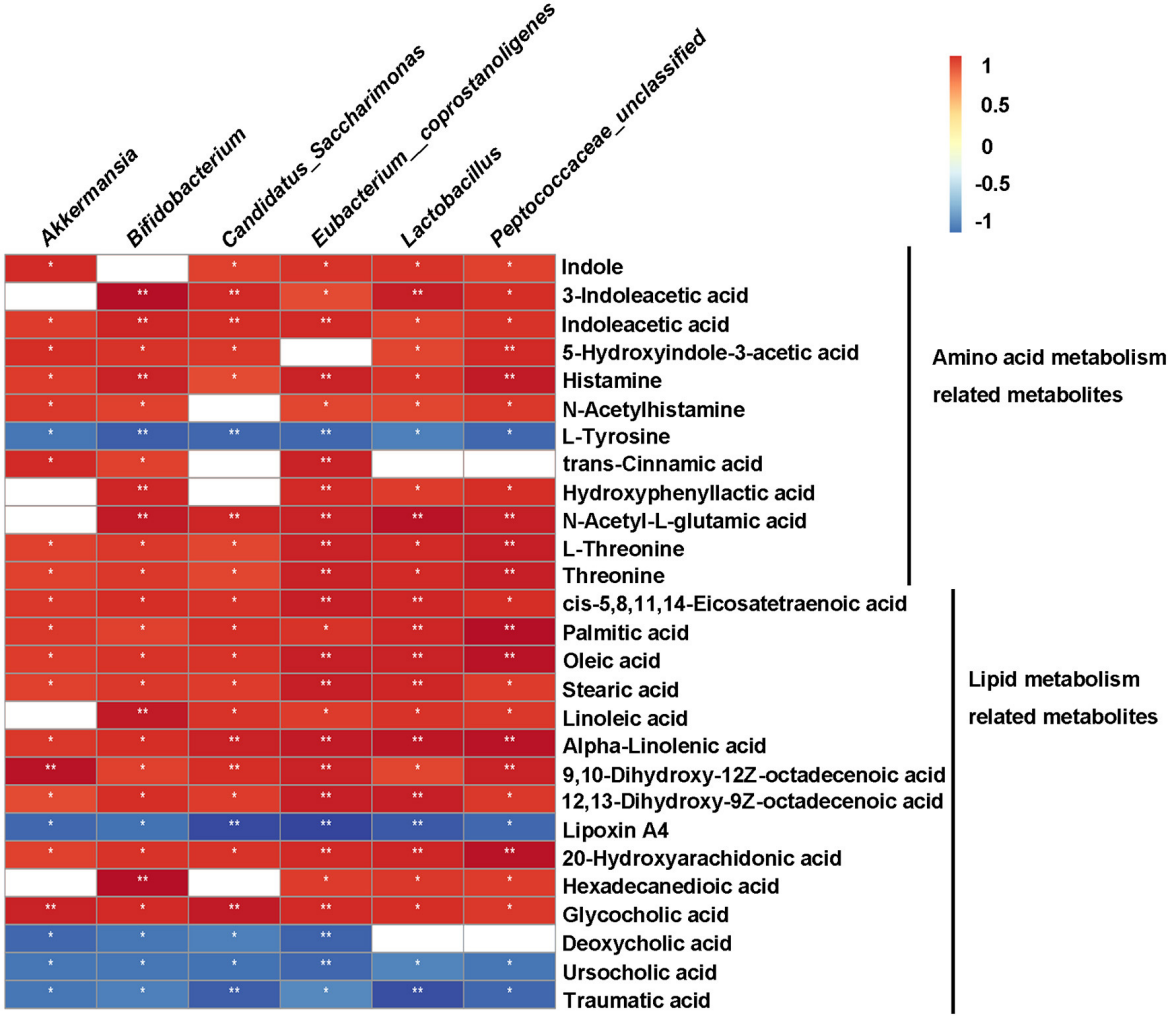
Correlation between metabolites and bacteria (genus-level) enriched by WGQK. Red indicates positive correlation and black indicates negative correlation, the darker the color, the stronger the correlation. *p*-values of correlations were adjusted by false discovery rate (FDR). *p < 0.05, **p < 0.01.

To clarify the relationship between gut microbiota and metabolites, we performed Spearman correlation analysis between the significant differential genera and metabolites (Kruskal-Wallis test, *p* < 0.05, ratio ≥2 or ≤ 0.5, and VIP ≥ 1) of HFD vs. HFD+QK, with a standard of *p* < 0.05, *R* > 0.9. A total of 55 metabolites were predicted to positively correlate with specific genera, while 93 metabolites were found to negatively correlate with specific genera (Supplementary Dataset S1). We further investigated the correlation of the six genera enriched by WGQK (based on LefSE analysis results) and the metabolites related to the amino acid and lipid metabolism pathways (Figure 8). Among the metabolites involved in the amino acid metabolism pathway, all metabolites were positively correlated with the six genera, except for L-tyrosine. In terms of lipid metabolism, the unsaturated fatty acids (oleic acid, 9,10-dihydroxy-12Z-octadecenoic acid, and 12,13-dihydroxy-9Z-octadecenoic acidα-linolenic acid) were positively correlated with the genera. In contrast, low levels of secondary bile acids (deoxycholic acid and ursocholic acid) and traumatic acid were observed in the HDF+QK group, which had a significant negative correlation with the six genera (Figure 8).

### WGQK Affected the Lipid Metabolism and FXR-FGF15 Signal

**Figure 9:**
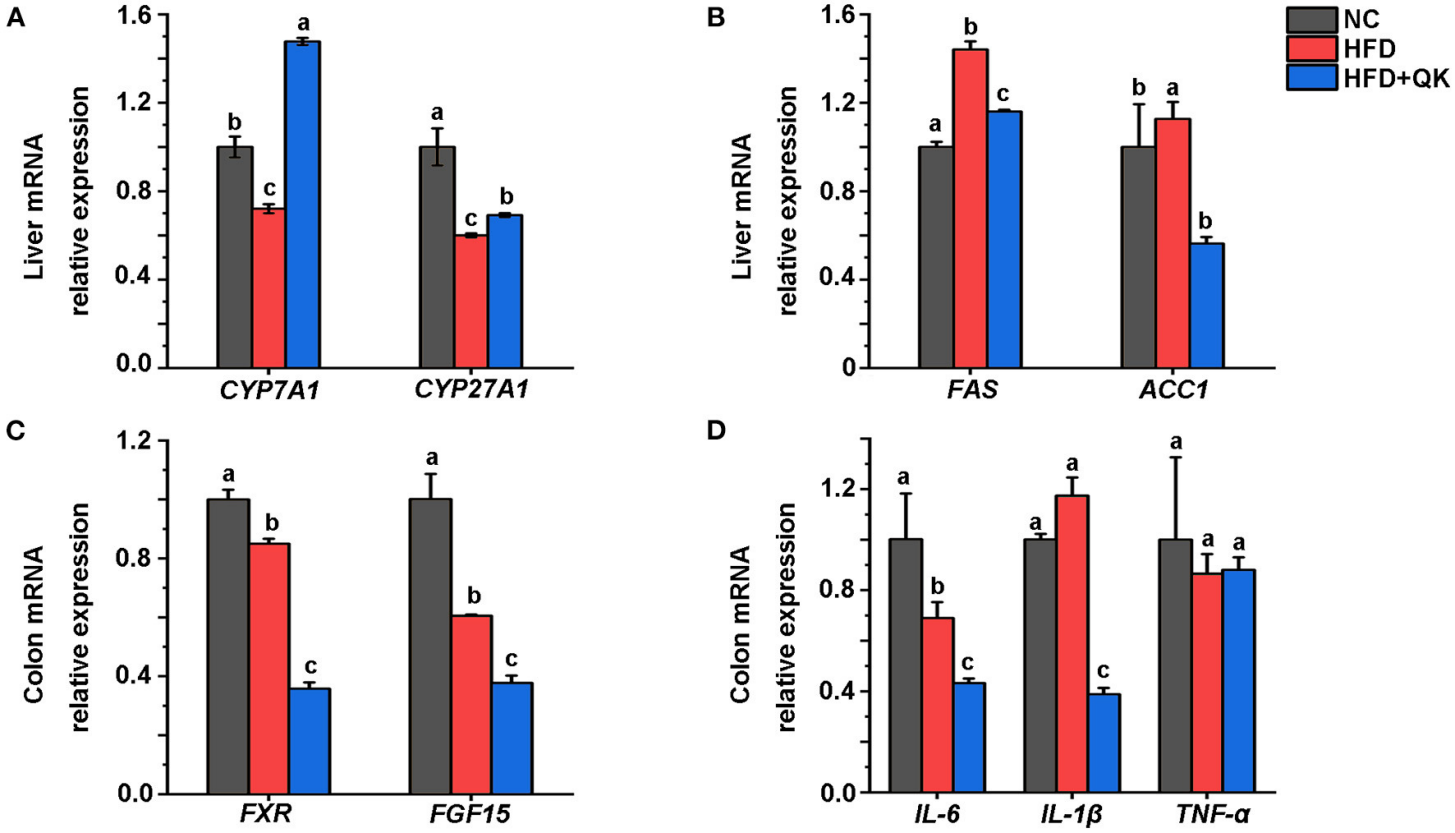
WGQK affects the liver lipid metabolism and intestinal function. **(A)** Relative mRNA expression of BA synthesis genes CYP7A1 and CYP27A in the liver, **(B)** Relative mRNA expression of Lipogenesis genes *FAS* and *ACC1* in the liver; **(C)** Relative mRNA expression of *FXR* and *FGF15* in the colon, **(D)** Expression of three inflammation genes in the colon. Data are presented as mean ± SD (*n* = 3), significant difference was determined using an unpaired two-tailed *t*-test, different superscript letters of any two means represent significant differences (*p* < 0.05).

To investigate the mechanism underlying the anti-obesity effects of WGQK, we detected the relative expression levels of some key genes might participate in regulating host lipid metabolism. The enzyme cholesterol 7α-hydroxylase (CYP7A1) and mitochondrial steroid 27-hydroxylase (CYP27A1) regulate the pathway of primary bile acids production from cholesterol. We found that the mRNA expression level of *CYP7A1*, but not *CYP27A1*, was significantly increased by WGQK, although both genes were inhibited in the HFD group (Figure 9A). Fatty acid synthase (Fas) and acetyl-CoA carboxylase (Acc1) are two important enzymes associated with lipogenesis (29). The relative expression of *FAS* and *ACC1* was found to be decreased in the HFD+QK group compared with the HFD group (Figure 9B).

The nuclear bile acid receptor farnesoid X receptor (FXR) and its downstream gene *FGF15* in the intestinal are thought to play critical roles in regulating glucose, lipid, and energy metabolism (30). We found that WGQK inhibited the mRNA expression of *FXR* and *FGF15* (Figure 9C). Also, WGQK could reduce the mRNA expression of genes encoding proinflammatory factors IL-6 and IL-1β (Figure 9D). These data indicated that WGQK might increase cholesterol catabolism into bile acids and suppress lipogenesis in liver.

## Discussion

In the current study, we evaluated the effects of WGQK intake on high-fat diet-induced obese mice. We found that WGQK reduced body weight gain, serum lipid level, and AST. It also improved glucose tolerance and hepatocyte degeneration induced by a high-fat diet. Through integrated analysis of gut microbiota and fecal metabolome data, we identified some specific gut bacterial species and metabolites that benefit from the WGQK intervention, which might contribute to the improved metabolic outcomes.

The results of gut microbiome analysis suggested that the alpha diversity index of the HFD group was lower than that of the NC group, but was higher than that of the HDF+QK group (Figure 3). A previous study that used WGQK (Zangqing 320) as a food intervention in rats under HFD reported similar results (9). This demonstrates that WGQK reduces the richness and diversity of the gut microbiota.

Taxonomic analysis showed that Firmicutes was the most dominant phylum in all the groups. Furthermore, the relative proportions of Firmicutes and Bacteroidetes in the HFD+QK group were lower than those in the HDF group (Figure 4). A study has reported that “overnutrition” is associated with proportionally more Firmicutes and fewer Bacteroidetes (16). We propose that the reduction in the abundance of the phylum Firmicutes is more likely responsible for the anti-obesity effect of WGQK.

Interestingly, the abundance of the phylum *Verrucomicrobia* was significantly enriched by WGQK in mice under WGQK, which was also reflected by a substantial increase in the abundance of the genus *Akkermansia* (Figure 4). We found that WGQK enriched two strains of *Akkermansia, Akkermansiamuciniphila* and *uncultured Akkermansia* (Supplementary Figure 4). *Akkermansia muciniphila* has been identified as a dominant human bacterium that abundantly colonizes nutrient-rich environments and is inversely correlated with body weight in rodents and humans (31). Furthermore, *Akkermansia muciniphila* treatment reversed high-fat diet-induced metabolic disorders, including fat mass gain, metabolic endotoxemia, adipose tissue inflammation, and insulin resistance (17). Similar results were obtained through a proof-of-concept study in overweight and obese human volunteers, showing that supplementation with *A. muciniphila* was safe, well-tolerated, and improved several metabolic parameters (32). Thus, we speculate that these two strains of *Akkermansia* might be involved in the anti-obesity effects of WGQK.

In addition to *Akkermansia*, we found that *Bifidobacterium, Lactobacillus, Eubacterium_coprostanoligenes*, and *Candidatus_Saccharimonas* were increased by the WGQK intervention (Figure 5). Some of these bacteria, including lactic acid bacteria such as Lactobacillus and *Bifidobacterium*, are generally recognized as the best-characterized probiotics (33). *Bifidobacterium* has been found to reduce body fat weight and blood serum levels (TC, HDL-C, LDL-C, AST, and ALT) in rats (18), while *Lactobacillus plantarum CQPC01* slowed the HFD-induced increase in body weight, decreased the organ indices, alleviated hepatic lipid accumulation, and inhibited the increased adipose cell volume (19). Feeding *Lactobacillus casei NCDC 19* in high-fat diet-induced obese mice has been reported to reduce body weight gain, epididymal fat weights, blood glucose, and plasma lipids (34). In addition, *g. Streptococcus* and *g. Eubacterium_coprostanoligenes* are thought to be two hub genera in the fecal micro-ecosystem under HFD and to mediate the effect of HFD on dyslipidemia through sphingosine (35). Overall, our results suggest that WGQK intervention selectively enriches a group of gut bacteria, which might contribute to its anti-obesity and anti-hyperlipidemic effects.

Through metabolome analysis, we identified several potential fecal biomarkers from the significant differential metabolites among the three groups. Four metabolites [indole, 3-indoleacetic acid, indole acetic acid (IAA), and 5-hydroxyindole-3- acetic acid] that are the products of the bacteria metabolizing tryptophan, were significantly enhanced in the HFD+QK group. They were predicted to be negatively correlated with body weight gain, liver function damage, and glucose tolerance (Figure 7). A recent study reported that tryptophan-derived bacterial metabolites could reduce body weight gain in rats (36), enhance the intestinal epithelial barrier, and attenuate indicators of inflammation (37). Our correlation analysis results showed that these tryptophan metabolites were positively correlated with *Lactobacillus, Peptococcaceae_unclassified*, and *Bifidobacterium* (Figure 8). Previous studies have confirmed the capacity of these genera to degrade tryptophan. *Lactobacillus* has been found to convert tryptophan to indole aldehyde (IAld) and ILA *via* the aromatic amino acid aminotransferase (ArAT) and indole lactic acid dehydrogenase (ILDH) (38, 39). ILDH can also convert tryptophan to indole acrylic acid (IA) and IPA (40). *Bifidobacterium* has been reported to convert tryptophan to ILA (41). Another possibility to link gut microbiota with Trp metabolism might be *via* Indoleamine 2,3-dioxygenase 1 (IDO1). IDO1 has been reported to regulate the Kynurenine pathway in Trp catabolism. Its expression can be negatively regulated by butyrate, a product of fermentation of dietary fibers by the gut microbiota (42). We found that the mRNA expression of Indoleamine 2,3-dioxygenase 1 (IDO1) was reduced in the colon in the HFD+QK group (Supplementary Figure 6). The decrease of IDO1 is predicted to increase the alternative Indole pathway *via* gut microbiota (43), which was consistent with our metabolome data. Overall, these results indicated that WGQK might increase the products of tryptophan catabolism by gut microbiota, such as indole and its derivatives, to attenuate intestinal inflammation and obesity.

Another interesting finding was the presence of histamine, a histidine metabolite. Its abundance in the HFD+QK group was 13 times higher than that in the other groups (Figure 6), and was predicted to be negatively correlated with body weight gain and AUC of GTT (Figure 7). Histamine is a neurotransmitter in the brain and plays a pivotal role in various physiological functions, such as feeding behavior and energy homeostasis. The mice with a disrupted histidine decarboxylase (HDC) gene, encoding a rate-limiting enzyme in histamine synthesis pathway, was found to be prone to obesity under high-fat diet (44). Evidence is emerging that activation of histamine signaling in the hypothalamus might have substantial anti-obesity and antidiabetic functions, and hypothalamic histamine H1 and H3 receptors are involved in the regulation of food rhythm, leptin resistance, and diabetes (44, 45).

In terms of lipid metabolism, we found that WGQK had a profound impact on the biosynthesis of unsaturated fatty acids and increased the levels of monounsaturated fatty acids (MUFAs), such as oleic acid, 9,10-dihydroxy-12Z-octadecenoic acid, and polyunsaturated fatty acids (PUFA)α-linolenic acid in feces (Figure 6). MUFA has been shown to have a beneficial effect in regulating body weight (46). Liu et al. found that high oleic canola oil could reduce fat mass and prevent metabolic syndrome (47). α-linolenic acid is a member of the omega-3 PUFA family, which has been reported to reduce the risk of cardiovascular disease (48). Meanwhile, several reports have shown that α-linolenic acid could alleviate obesity and reduce the levels of inflammatory markers, such as serum insulin and leptin, and improve cholesterol homeostasis (49, 50). In addition, α-linolenic acid could restore the HFD-induced gut microbial community structure and composition shifts (51); similar results were observed in our study (Figure 5A). Meanwhile, correlations between gut bacteria (genus level) enriched by WGQK and these unsaturated fatty acids showed significant and positive correlations (Figure 8).

Furthermore, we found that the intestinal expression of *FXR* and *FGF15* was reduced in parallel with an increase in gene expression for CYP7A1 in the HFD+QK group (Figure 9). FXR is a nuclear receptor that regulates the homeostasis of BAs, lipids and glucose (30). The activation of intestinal FXR promotes fibroblast growth factor 15 (FGF15), resulting in the inhibition of hepatic BA biosynthesis from cholesterol (52). Emerging data showed that inhibiting ileal FXR-FGF15 induced beneficial effects alleviating non-alcoholic fatty liver disease (NAFLD), obesity, and insulin resistance (53, 54). An animal and human trial on the cholesterol-lowering effect of theabrownin showed that the inhibition of intestinal FXR-FGF15 signaling resulted in increased hepatic production and fecal excretion of BAs (55). Transgenic mice overexpressing *CYP7A1* has been found to be resistant to high-fat diet-induced obesity, fatty liver, and insulin resistance (56). Therefore, we proposed that WGQK might reduce cholesterol by inhibiting the intestinal FXR-FGF15 signaling pathway. Further detection of hepatic BA composition will reveal how the interaction of FXR signaling and BA synthesis modulate the cholesterol level in the liver.

Another interesting finding was the presence of succinate, which had a higher abundance in the HFD + QK group than in the HFD group. *Prevotella* is regarded as a succinate producer and could improve glucose homeostasis *via* intestinal gluconeogenesis (IGN) (57). The relative abundance of *Prevotella* was found to be higher in the HFD+QK group than in the HFD group (Supplementary Figure 5), supporting their possible roles in host health.

In summary, WGQK intervention improved HFD-induced obesity, and its potential mechanism might involve the following: (1) selectively enriched a group of gut bacteria which have been demonstrated to alleviate obesity, including *Akkermansia, Lactobacillus*, and *Bifidobacterium*; (2) increased accumulation of indole and its derivatives, which are the products of tryptophan catabolism by gut microbiota; (3) through the beneficial effects of unsaturated fatty acids; (4) inhibition of the intestinal FXR-FGF15 signaling pathway to regulate bile acids production. Further studies using antibiotic-treated or germ-free mice and fecal microbiota transplantation will help us better understand better the effects of WGQK depending on gut microbiota and the interaction of metabolites-microbial on host metabolic health.

## Data Availability Statement

The 16S rRNA gene sequences were provided and available at NCBI Sequence Read Archive (SRP) repository with Accession Code PRJNA757532; untargeted metabolomic data have been deposited to the EMBL- EBI MetaboLights database with the identifier MTBLS3354, the complete data set can be accessed at www.ebi.ac.uk/metabolights/MTBLS3354.

## Ethics Statement

The animal study was reviewed and approved by the Institutional Animal Care and Use Committee of the Laboratory Animals Center at Zhejiang University, Hangzhou, China (No. ZJU20210160).

## Author Contributions

NH and WL designed the study. XL and JS conducted the animal trial, sample collection, and physical analysis. XL and XH performed the bioinformatics analysis of 16S rRNA sequencing and untargeted metabolomics data. XL and NH wrote the manuscript. HB, WL, MZ, and HD contributed to the discussion of the work and assisted in drafting the manuscript. All authors read and approved the final manuscript.

## Funding

This work was supported by the China Agriculture Research System (CARS-05-05A) and the Chinese Academy of Engineering Academy-Locality Cooperation Project (2019-ZJ-JS-02), and the Key Science Technology Project of Medicine and Health, Zhejiang Province, Foundation of Scientific Research of National Health Care Commission (WKJ-ZJ-2009).

## Conflict of Interest

The authors declare that the research was conducted in the absence of any commercial or financial relationships that could be construed as a potential conflict of interest.

## Publisher's Note

All claims expressed in this article are solely those of the authors and do not necessarily represent those of their affiliated organizations, or those of the publisher, the editors and the reviewers. Any product that may be evaluated in this article, or claim that may be made by its manufacturer, is not guaranteed or endorsed by the publisher.

## Supplementary Material

Supplementary Figure 1 Boxplot of significant (*p* < 0.05) differential gut bacteria (genus level) of HFD vs. NC **(A)**, and HFD vs. HFD+QK **(B)**.

Supplementary Figure 2 Significant differential metabolites in NC vs. HFD, and HFD vs. HFD+QK analysis. **(A,B)** PLS-DA score plots analysis of NC vs. HFD, and HFD vs. HFD+QK; **(C,D)** volcano plot with a standard of ratio ≥2 or ≤0.5 and VIP ≥ 1, significant differential metabolic ions were shown as red (up) or green (down); **(E,F)** The abundance of significant differential metabolites which had opposite regulation in NC vs. HFD and HFD vs. HFD+QK. Data are presented as mean ± SD (*n* = 5), significant difference was determined using an unpaired two-tailed *t*-test, different superscript letters of any two means represent significant differences (*p* < 0.05).

Supplementary Figure 3 Correlation between significant differential metabolites which had opposite regulation and the host obesity parameters. Red indicates positive correlation and black indicates negative correlation, the darker the color, the stronger the correlation, ^*^*p* < 0.05, ^**^*p* < 0.01.

Supplementary Figure 4 Relative abundance of two strains of Akkermansia. **(A)** Akkermansia_muciniphila; **(B)** uncultured_Akkermansia_sp. Data are presented as mean ± SD (*n* = 5), significant difference was determined using an unpaired two-tailed *t*-test, different superscript letters of any two means represent significant differences (*p* < 0.05).

Supplementary Figure 5 **(A)** Relative abundance of Prevotella in the HFD and HFD+QK groups; **(B)** Relative abundance of succinic acid in the HFD and HFD+QK groups. Data are presented as mean ± SD (*n* = 5), significant difference was determined using an unpaired two-tailed *t*-test, different superscript letters of any two means represent significant differences (*p* < 0.05).

Supplementary Figure 6 Relative expression of Indoleamine 2,3-dioxygenase 1 (IDO1) in the colon. Data are presented as mean ± SD (*n* = 5). Significant difference was determined using an unpaired two-tailed *t*-test, different superscript letters of any two means represent significant differences (*p* < 0.05).

Supplementary Table S1 Primer sequence information of the qRT-PCR.

Supplementary Data Set 1 Spearman correlation between the significant differential genera and metabolites.
