# Supplementary material for: Whole Grain Qingke Attenuates High-Fat Diet-Induced Obesity in Mice With Alterations in Gut Microbiota and Metabolite Profile
Source: Front Nutr. 2021 Dec 7;8:761727. doi: 10.3389/fnut.2021.761727 (PMC8688713; doi:10.3389/fnut.2021.761727)
Supplement: Supplementary Figure 1 — Boxplot of significant (p < 0.05) differential gut bacteria (genus level) of HFD vs. NC (A), and HFD vs. HFD+QK (B). [file Data_Sheet_1.PDF]

# *Supplementary Material*

Supplementary Figures

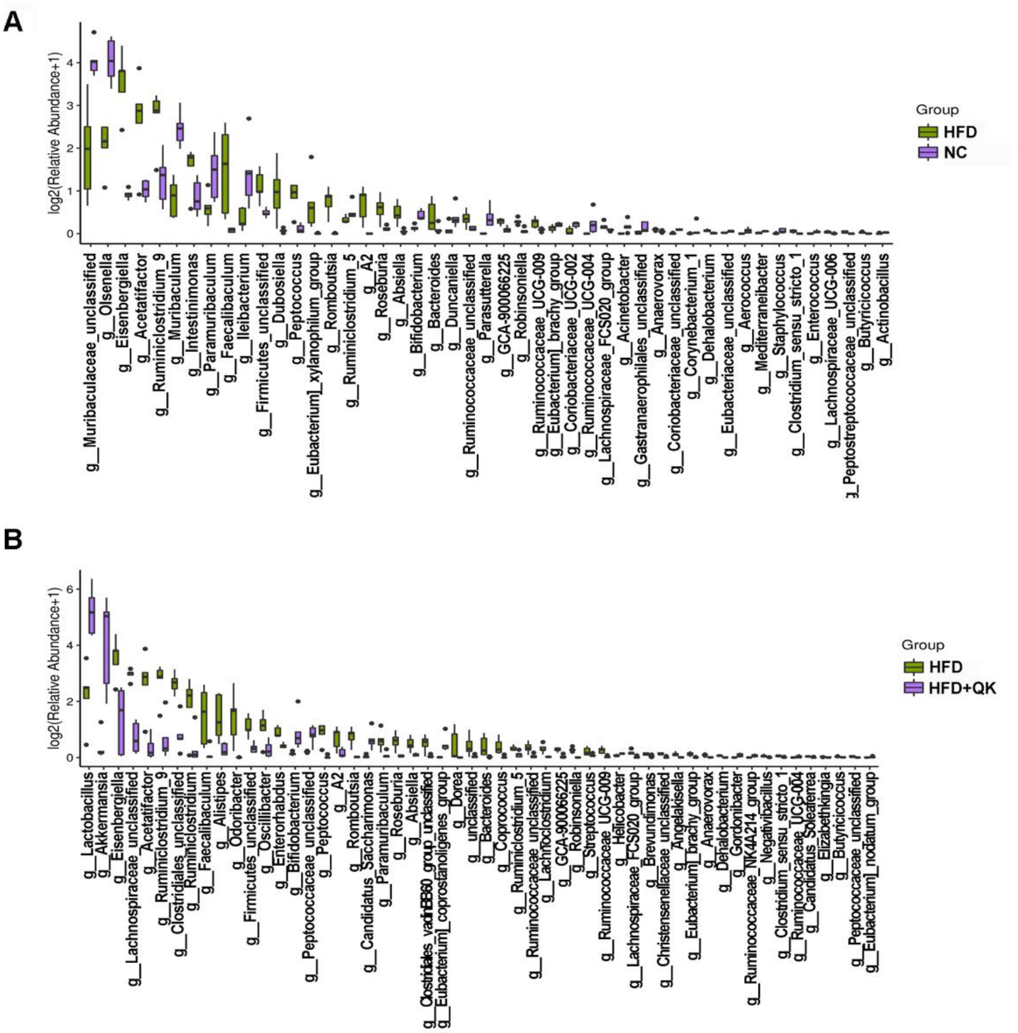


**Supplementary Figures 1.** Boxplot of significant ($p<0.05$) differential gut bacteria (genus level) of HFD versus NC (A), and HFD versus HFD+QK (B).

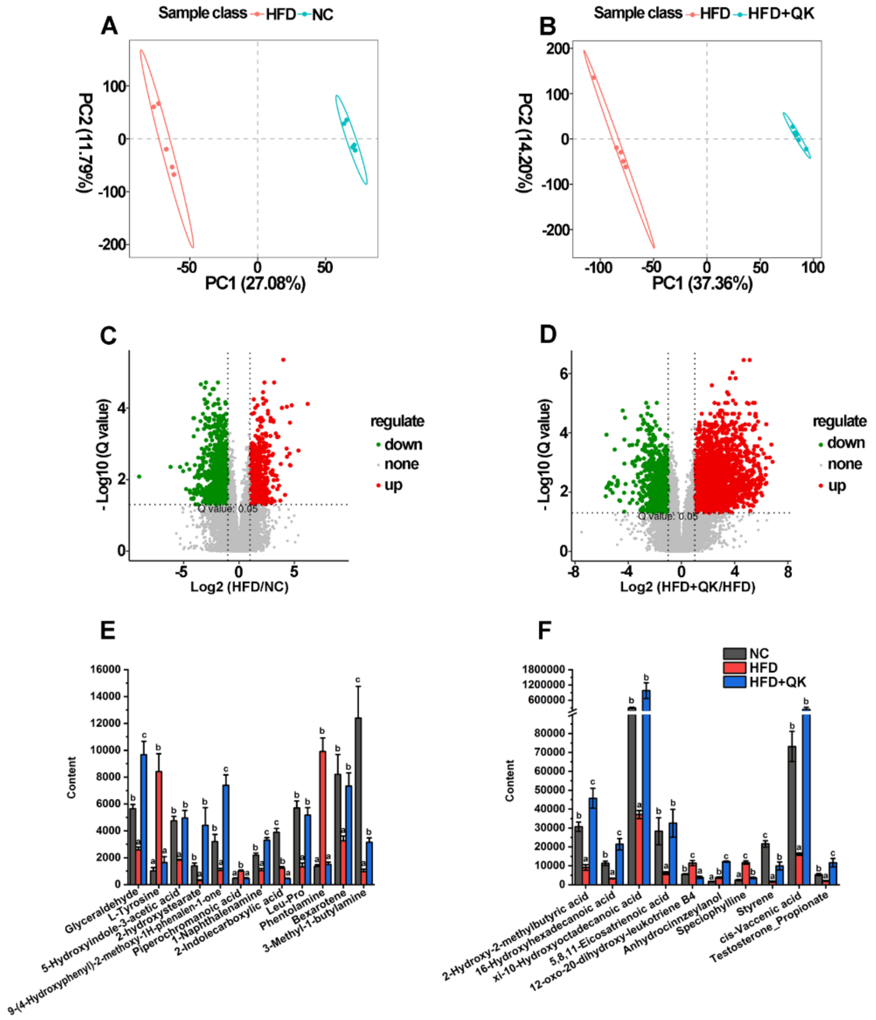


**Supplementary Figures 2.** Significant differential metabolites in NC versus HFD, and HFD versus HFD+QK analysis. (A, B) PLS-DA score plots analysis of NC versus HFD, and HFD versus HFD+QK; (C, D) volcano plot with a standard of ratio≥2 or ≤0.5 and VIP≥1, significant differential metabolic ions were shown as red (up) or green (down); (E, F) The abundance of significant differential metabolites which had opposite regulation in NC versus HFD and HFD versus HFD+QK. Data are presented as mean ± SD (n=5), significant difference was determined using an unpaired two-tailed t-test, different superscript letters of any two means represent significant differences ($p < 0.05$).

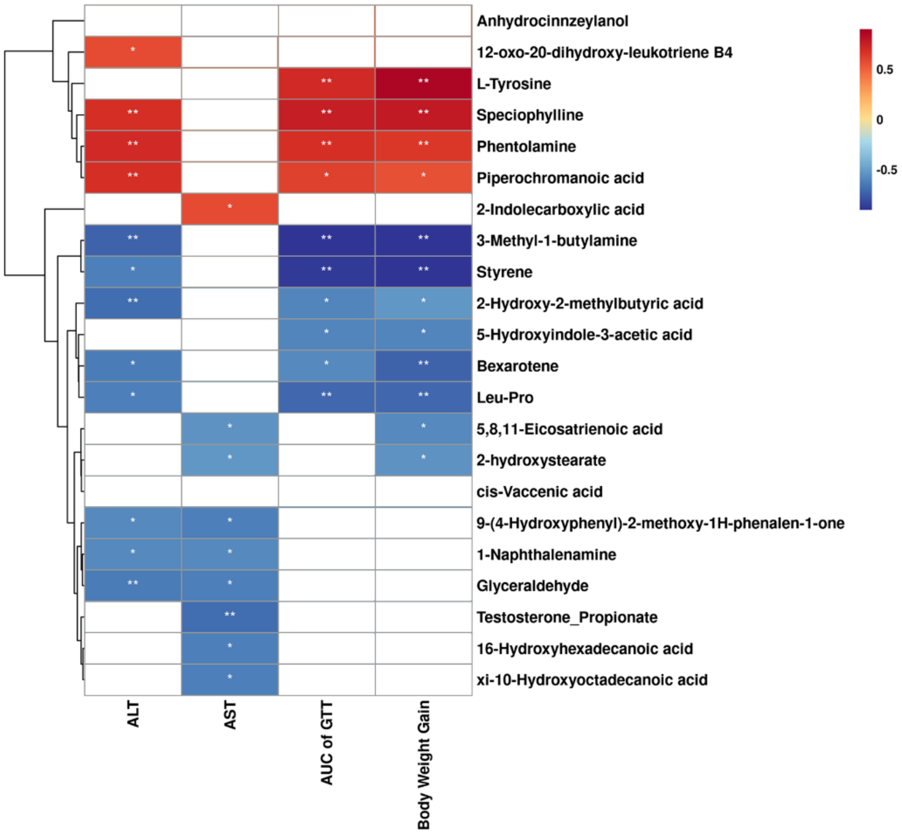


**Supplementary Figures 3.** Correlation between significant differential metabolites which had opposite regulation and the host obesity parameters. Red indicates positive correlation and black indicates negative correlation, the darker the color, the stronger the correlation, * $p<0.05$, * * $p<0.01$.

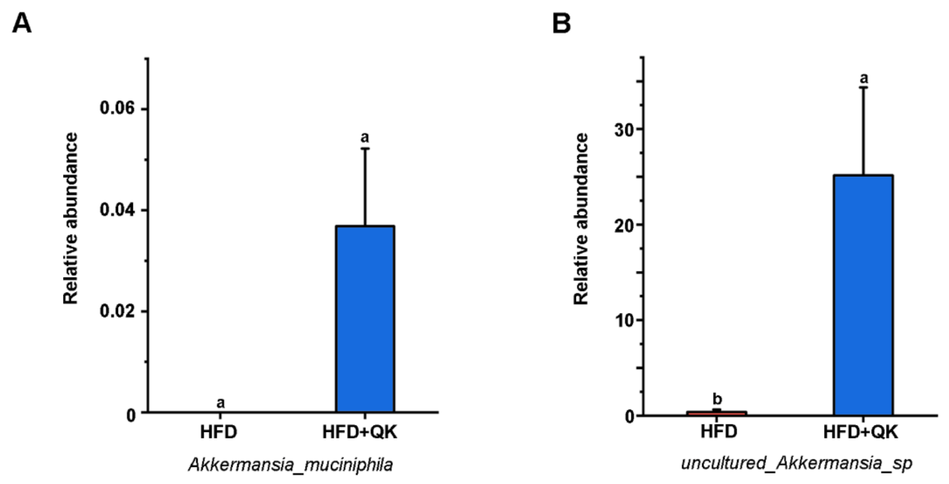


**Supplementary Figures 4.** Relative abundance of two strains of Akkermansia. (A) *Akkermansia_muciniphila*; (B) *uncultured_Akkermansia_sp*. Data are presented as mean ± SD (n=5), significant difference was determined using an unpaired two-tailed t-test, different superscript letters of any two means represent significant differences ($p$ < 0.05).

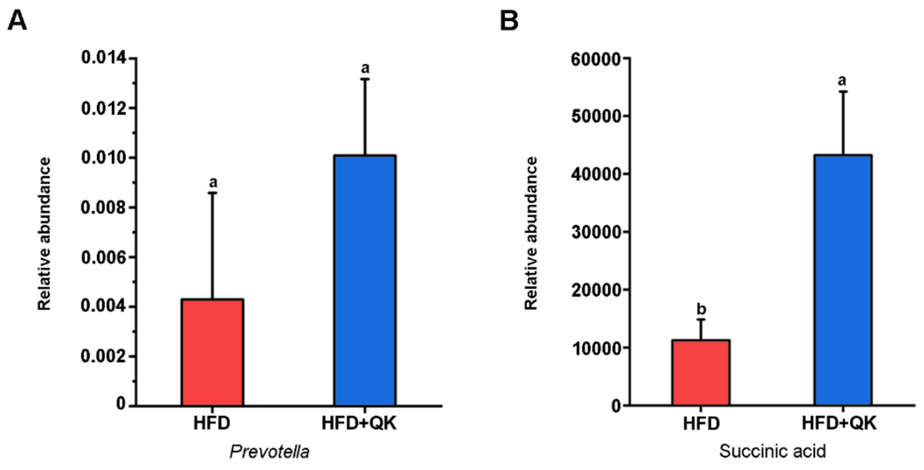


**Supplementary Figures 5.** (A) Relative abundance of *Prevotella* in the HFD and HFD+QK groups; (B) Relative abundance of succinic acid in the HFD and HFD+QK groups. Data are presented as mean ± SD (n=5), significant difference was determined using an unpaired two-tailed *t*-test, different superscript letters of any two means represent significant differences ($p < 0.05$).

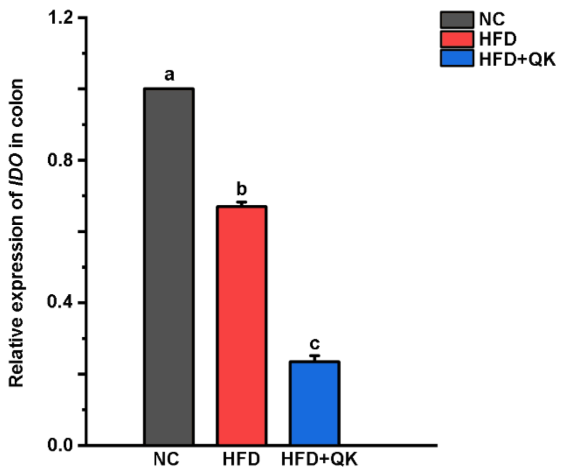


**Supplementary Figure 6.** Relative expression of Indoleamine 2,3-dioxygenase 1 (IDO1) in the colon. Data are presented as mean ± SD (n=5). Significant difference was determined using an unpaired two-tailed t-test, different superscript letters of any two means represent significant differences ($p < 0.05$).

**Table S1.** Primer sequence information of the qRT-PCR.

| Primer | Forward sequence | Reverse sequence |
|---|---|---|
| CYP7A1 | GGGCATCTCAAGCAAACACC | AATGGCATTCCCTCCAGAGC |
| CYP27A1 | TGAACGAGTACCACACCAGG | CATCAGACTATGGCGCAGGT |
| FAS | CTTGGGTGCTGACTACAACC | GCCCTCCCGTACACTCACTC |
| ACC1 | CTGTGCCAACCACAAGATGC | TCAGGTCTGAGTGCCGGATA |
| FXR | CTGACCCTGCCATCCAAGT | TCAGAAGCCGATGTTCCTCT |
| FGF15 | GATTGCCATCAAGGACGTCAG | TCAGCCCGTATATCTTGCCG |
| IL-6 | GTTCTCTGGGAAATCGTGGA | TGTACTCCAGGTAGCTA |
| IL-1 β | TCCATGAGCTTTGTACAAGGA | AGCCCATACTTTAGGAAGACA |
| TNF- α | AGACCCTCACACTCAGATCA | TCTTTGAGATCCATGCCGTTG |
| IDO1 | CAATCAAAGCAATCCCCACTG TATC | ATATATGCGGAGAACGTGGAAAAA C |
| 18S rRNA | GGACACGGACAGGATTGACA | GACATCTAAGGGCATCACAG |
